# Supplementary material for: Nematophagous fungus Arthrobotrys oligospora mimics olfactory cues of sex and food to lure its nematode prey
Source: eLife. 2017 Jan 18;6:e20023. doi: 10.7554/eLife.20023 (PMC5243009; doi:10.7554/eLife.20023)
Supplement: Supplementary file 2. — DOI: http://dx.doi.org/10.7554/eLife.20023.017 [file elife-20023-supp2.docx]

**Supplementary file 2A: Numbers of filtered and mapped reads in RNA-seq data sets.**

| **Input data**  **(post-filter)** | **Total reads** | **Aligned 0 times** | **Aligned exactly 1 time** | **Aligned 2+ times** | **Overall alignment rate** |
| --- | --- | --- | --- | --- | --- |
| AWC (all data, pooled) | 131,614,334 | 109,188,636 (82.96%) | 10,998,887 (8.36%) | 11,426,811 (8.68%) | 17.04% |
| Larvae (all data, pooled) | 23,369,056 | 5,513,350 (23.59%) | 7,422,251 (31.76%) | 10,433,455 (44.65%) | 76.41% |
| AWC set 1 (cells 1, 2, 4) | 45,743,930 | 39,323,424 (85.96%) | 3,080,637 (6.73%) | 3,339,869 (7.30%) | 14.04% |
| AWC set 2 (cells 3, 5) | 50,689,999 | 43,433,989 (85.69%) | 3,535,163 (6.97%) | 3,720,847 (7.34%) | 14.31% |
| Larvae pool 1 | 14,756,459 | 4,465,670 (30.26%) | 4,490,260 (30.43%) | 5,800,529 (39.31%) | 69.74% |
| Larvae pool 2 | 8,612,597 | 1,047,680 (12.16%) | 2,931,991 (34.04%) | 4,632,926 (53.79%) | 87.84% |
| AWC cell 1 | 12,022,887 | 10,849,959 (90.24%) | 494,185 (4.11%) | 678,743 (5.65%) | 9.76% |
| AWC cell 2 | 18,381,663 | 15,919,608 (86.61%) | 1,211,992 (6.59%) | 1,250,063 (6.80%) | 13.39% |
| AWC cell 3 | 16,081,298 | 13,807,723 (85.86%) | 1,159,038 (7.21%) | 1,114,537 (6.93%) | 14.14% |
| AWC cell 4 | 15,339,380 | 12,553,857 (81.84%) | 1,374,460 (8.96%) | 1,411,063 (9.20%) | 18.16% |
| AWC cell 5 | 34,608,701 | 29,626,266 (85.60%) | 2,376,125 (6.87%) | 2,606,310 (7.53%) | 14.40% |

"Total reads" denotes the complete set of quality-filtered reads for a given data set that were mapped with RSEM to a *C. elegans* gene index in order to compute gene expression values. With RSEM, a read can be mapped to the gene index either 0 times (i.e., it can fail to map at all); it can map exactly 1 time (i.e., it can map to a unique site in the gene index); or it can map 2+ times. For each RNA-seq data set, the numbers and percentages of reads with each status are given, as is the overall percentage of reads that mapped to the gene index. Filtered reads from five independent AWC RNA-seq data sets were individually mapped; their results are given in Supplementary Data Set S1, though these RSEM analyses were not used for bulk expression data or statistical analysis. The set of "AWC (all data, pooled)" data includes 35,180,405 reads from a set of aliquots from 9 independent RT-PCR products from 9 individual AWC neurons, that were aliquotted and mixed together before being sequenced; since these 35.1M reads could not be disentangled into sequences of independent origin, they were not included in AWC sets 1 and 2. The reads for these AWC sets were mixed in silico from the readsets of individual AWC cells before their RSEM analysis. RSEM results from these AWC sets, and from larval pools 1 and 2, were used for statistical analysis of differential gene expression with DESeq2.

**Supplementary file 2B: Numbers of genes with above-background expression in AWC data sets.**

| **Data set** | **Expressed genes** | **Protein-coding** | **ncRNA** | **Housekeeping** | **GPCR** |
| --- | --- | --- | --- | --- | --- |
| AWC (all  data, pooled) | 5,326 | 5,294 | 32 | 984 | 32 |
| AWC cell 1 | 3,341 | 3,323 | 18 | 711 | 46 |
| AWC cell 2 | 2,946 | 2,928 | 18 | 693 | 19 |
| AWC cell 3 | 2,628 | 2,615 | 13 | 667 | 16 |
| AWC cell 4 | 3,528 | 3,499 | 29 | 808 | 15 |
| AWC cell 5 | 2,769 | 2,757 | 12 | 711 | 11 |
| AWC, any | 5,937 | 5,894 | 43 | 1,010 | 48 |

Genes are counted as expressed if, in a given RSEM analysis of a given data set, the gene has a minimum expression value (minTPM) of ≥ 0.1 TPM in a 99% confidence interval. The six discrete data sets (pooled, and cells 1-5) are named as in Supplementary File 1A. For "AWC, any", genes are counted as expressed if they have minTPM ≥ 0.1 in any of the six discrete sets. Although most gene expression is successfully detected by RSEM analysis of all pooled AWC data, a minority of genes are only detected in RSEM analyses of one or more individual AWC cells. The overall AWC gene expression visualized in Figure 7 is from the "AWC (all data, pooled)" RSEM analysis.
